# Endonuclease-containing *Penelope* retrotransposons in the bdelloid rotifer *Adineta vaga* exhibit unusual structural features and play a role in expansion of host gene families

**DOI:** 10.1186/1759-8753-4-19

**Published:** 2013-08-27

**Authors:** Irina R Arkhipova, Irina A Yushenova, Fernando Rodriguez

**Affiliations:** 1 Josephine Bay Paul Center for Comparative Molecular Biology and Evolution, Marine Biological Laboratory, 7 MBL Street, Woods Hole, MA 02543, USA

**Keywords:** Retrotransposon, Reverse transcriptase, GIY-YIG endonuclease

## Abstract

**Background:**

*Penelope*-like elements (PLEs) are an enigmatic group of retroelements sharing a common ancestor with telomerase reverse transcriptases. In our previous studies, we identified endonuclease-deficient PLEs that are associated with telomeres in bdelloid rotifers, small freshwater invertebrates best known for their long-term asexuality and high foreign DNA content. Completion of the high-quality draft genome sequence of the bdelloid rotifer *Adineta vaga* provides us with the opportunity to examine its genomic transposable element (TE) content, as well as TE impact on genome function and evolution.

**Results:**

We performed an exhaustive search of the *A. vaga* genome assembly, aimed at identification of canonical PLEs combining both the reverse transcriptase (RT) and the GIY-YIG endonuclease (EN) domains. We find that the RT/EN-containing *Penelope* families co-exist in the *A. vaga* genome with the EN-deficient RT-containing *Athena* retroelements. Canonical PLEs are present at very low copy numbers, often as a single-copy, and there is no evidence that they might preferentially co-mobilize EN-deficient PLEs. We also find that *Penelope* elements can participate in expansion of *A. vaga* multigene families via *trans*-action of their enzymatic machinery, as evidenced by identification of intron-containing host genes framed by the *Penelope* terminal repeats and characteristic target-site duplications generated upon insertion. In addition, we find that *Penelope* open reading frames (ORFs) in several families have incorporated long stretches of coding sequence several hundred amino acids (aa) in length that are highly enriched in asparagine residues, a phenomenon not observed in other retrotransposons.

**Conclusions:**

Our results show that, despite their low abundance and low transcriptional activity in the *A. vaga* genome, endonuclease-containing *Penelope* elements can participate in expansion of host multigene families. We conclude that the terminal repeats represent the *cis*-acting sequences required for mobilization of the intervening region in *trans* by the *Penelope*-encoded enzymatic activities. We also hypothesize that the unusual capture of long N-rich segments by the *Penelope* ORF occurs as a consequence of peculiarities of its replication mechanism. These findings emphasize the unconventional nature of *Penelope* retrotransposons, which, in contrast to all other retrotransposon types, are capable of dispersing intron-containing genes, thereby questioning the validity of traditional estimates of gene retrocopies in PLE-containing eukaryotic genomes.

## Background

*Penelope*-like elements (PLEs) represent an ancient class of eukaryotic retroelements that shares a common ancestor with telomerase reverse transcriptases [1,2]. They can be found in protists, fungi, animals and plants, although their representation in these taxa can be very sporadic. The first *Penelope* element was identified in *Drosophila virilis,* where it was shown to participate in hybrid dysgenesis [3,4]. Its structural and functional properties are those of a typical retrotransposon, consisting of a single open reading frame (ORF). This ORF encodes a reverse transcriptase (RT) domain responsible for RNA-templated DNA synthesis, and an endonuclease (EN) domain responsible for integration of a reverse-transcribed cDNA copy into new chromosomal locations, generating short target site duplications (TSD) upon insertion. The EN domain associated with PLEs belongs to endonucleases of the GIY-YIG superfamily, which were originally identified in group I mobile introns from bacteria and organelles [5-8]. The *Penelope* EN domain was overexpressed in *E. coli* and shown to possess DNA cleavage activity, while the baculovirus-expressed RT was capable of RNA-dependent DNA synthesis *in vitro*[9].

In addition to EN-containing PLEs, there is a distinct PLE subclass which lacks the EN domain altogether [10]. Such EN-deficient PLEs are apparently able to prime DNA synthesis from the 3′ ends of the exposed chromosome termini, either at deprotected telomeres or at sites of double-strand DNA breakage, thereby obviating the need for endonuclease activity. Accordingly, their transposition is largely confined to chromosome ends, where it is followed by the addition of telomeric repeats to the truncated 5′ end of the element. Such EN-deficient PLEs are found in selected animal, fungal, protist and plant species, but their distribution is highly sporadic.

Rotifers of the class Bdelloidea, in which EN-deficient PLEs were discovered, are microscopic freshwater invertebrates that reproduce asexually, can survive frequent rounds of desiccation and rehydration, and contain significant amounts of horizontally transferred genes in their genomes [11-13]. The high-quality draft genome sequence of the first representative of the phylum Rotifera, the bdelloid *Adineta vaga*, was recently completed [14]. Only about 3% of its genomic DNA is represented by transposable elements (TEs), and while the diversity of families is high, each family contains very few members, indicating that incoming TEs do not proliferate efficiently in the *A. vaga* genome. While PLEs make up almost one-third of all *A. vaga* retroelements (a total of 24 families, occupying approximately 0.74 Mb of the 218-Mb assembly), the majority of the *A. vaga* PLEs are represented by EN-deficient *Athena* retroelements [10]. Here we report that the *A. vaga* genome also contains a small number of “canonical” *Penelope* elements with the GIY-YIG endonuclease domain, which, however, exhibit several highly unusual features.

## Results

### Identification and phylogenetic analysis of *A. vaga Penelope* families

**Figure 1:**
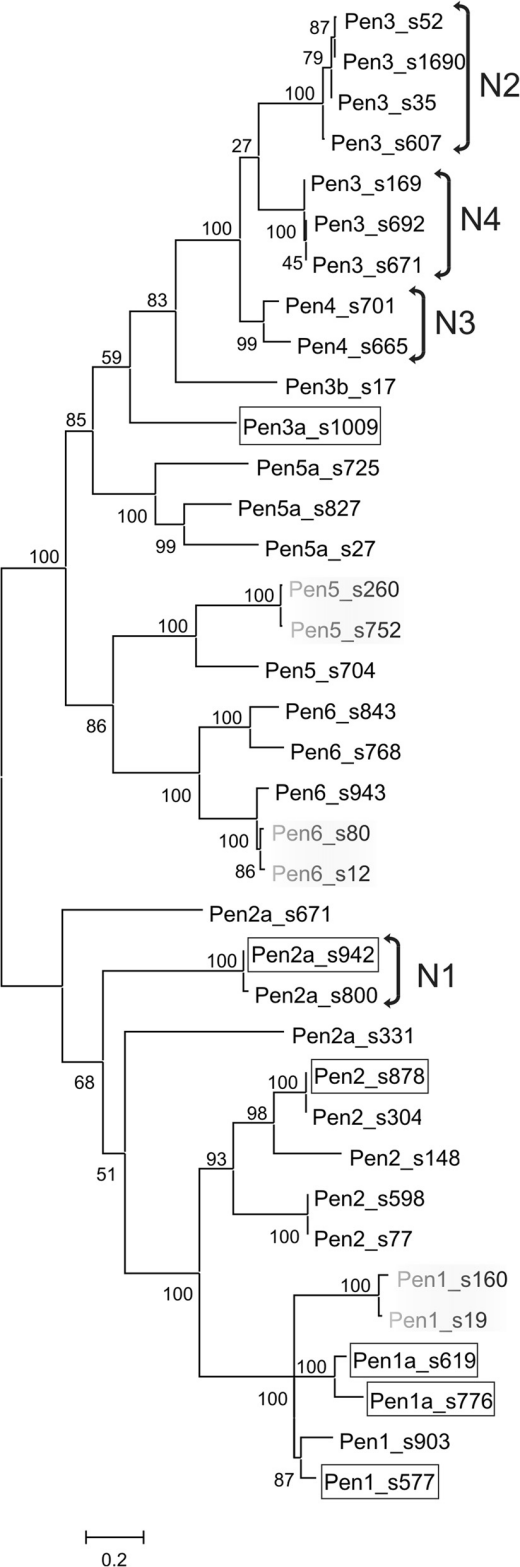
**Maximum likelihood analysis of*A. vaga Penelope*nucleotide sequences.** Branch support is indicated at the nodes. *Penelope* insertions localized in collinear allelic pairs are shaded. Intact copies are boxed. Brackets designated N1-N4 denote the presence of different N-rich inserts in each group of elements, as described in the text. Scale bar, nucleotide substitutions per site.

We performed an exhaustive search of the *A. vaga* genome assembly, aiming at identification of every *Penelope* copy in the assembly and reconstruction of their complete genomic evolutionary history. Additional file 1: Table S1 lists all of the full-length and partial copies, as well as their coordinates in the assembly. A total of 36 copies spanning a sufficiently long stretch (at least one-third) of the coding sequence to be included into phylogenetic analysis were aligned, and their phylogenetic relationships were determined in order to place their structural and functional properties into phylogenetic context (Figure 1). The corresponding alignment of *Penelope* coding sequences is provided in the Additional file 2: Data file 1. Based on their structural organization and the degree of protein sequence similarity, all *Penelope* retrotransposons in *A. vaga* could be divided into six major groups (*Pen1*-*Pen6_Av*) and further into 11 families (1a, 2b and so on), with each family represented by very few copies (Figure 1, Additional file 1: Table S1).

The evolutionary history of *Penelope* retrotransposons in the sequenced *A. vaga* isolate reveals that all copies are arranged in two major branches, consisting of *Pen1-2_Av* and *Pen3-6_Av* elements, respectively. High support values for the majority of nodes are indicative of relatively few insertion events that gave rise to extant copies. Overall, 6 out of 37 ORFs presented in Figure 1 appear intact (boxed), although intactness is not associated with a higher degree of proliferation in the genome. The majority of copies contain defects in their ORFs, such as frameshifts, in-frame stop codons, indels or truncations.

### Structural organization of *Penelope* families

**Figure 2:**
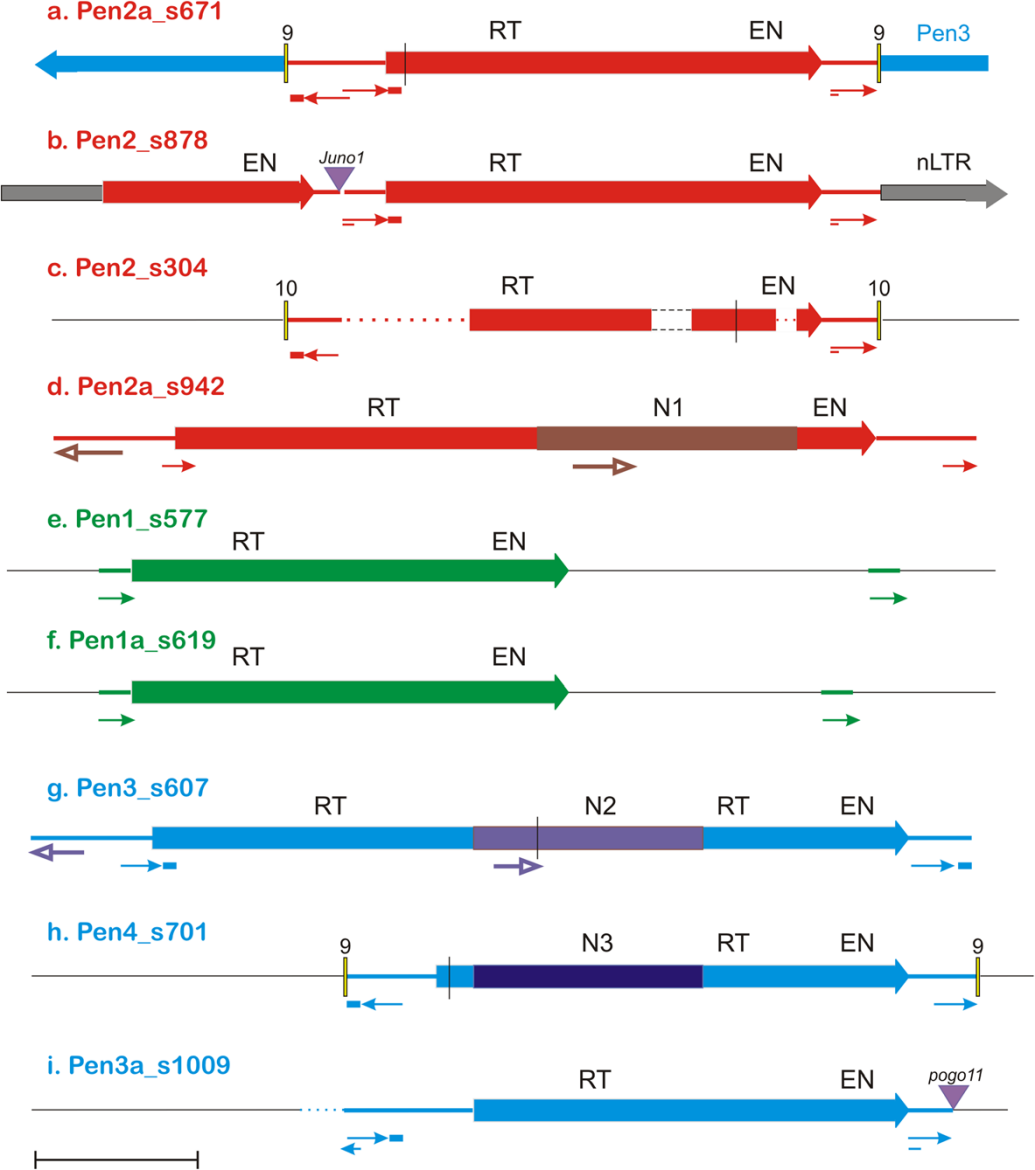
**Structural features of selected*A. vaga Penelope*retrotransposons.** ORFs are represented by colored boxes with arrows. RT, reverse transcriptase; EN, endonuclease. Thin arrows denote pLTRs, and small colored rectangles - its optional short extension (“tail”). Panels **(a-i)** correspond to different families. Thicker arrows represent repeats derived from sequences other than pLTRs. N1 to N3 denote different N-rich inserts within ORFs, shown by darker colors. Palindromes are shown by double lines; frameshifts or stop codons, by vertical lines; deletions, by dashed lines; gaps, by dotted lines. Numbers above small yellow boxes indicate the length of target site duplications in bp. Scale bar, 1 kb.

The typical *Penelope* structure, also observed in other animals, such as fruit flies and fish [3,15,16], is exemplified by a single-copy element *Pen2a_Av* appearing as a secondary insertion into *Pen3* on scaffold 671 in the opposite orientation (Figure 2a). Its 842-aa ORF, with an in-frame stop codon, is bounded by two 218-base pairs (bp) “pseudo-long terminal repeats” (pLTRs) [15,16], and an adjacent inverted pLTR completes the entire insert, which in this case is flanked by a 9-bp TSD. Also found is the characteristic 31-bp extension, or “tail”, which is optionally present at the 3′ end of one of the inverted pLTRs [16]. The pLTR includes the first 12 amino acids (aa) of the ORF, and together with the tail, this length can be extended to 22 amino acids. The inverted pLTR at the 5′ end overlaps the direct pLTR by 15 bp, and is 37-bp shorter than the two full pLTRs (181 bp). The pLTR contains a putative TATA box (TATATATA) separated by 20 bp from an initiator-(Inr)-like sequence (TCACT), and could, therefore, exhibit basal promoter activity. It is worth noting that this TATA sequence can be read in both directions, and the opposite direction also features a downstream Inr-like sequence ACATT, raising the possibility of a bidirectional promoter. *Pen2a_Av* occurs in the assembly only once, and has not given rise to any new copies or fragments. Similar pLTR structures are also found in most of the families described below.

For *Pen2_Av,* the only intact and potentially active ORF (838 aa) is present on scaffold 878 (Figure 2b). This element consists of two copies, one full-length and one 5′-truncated, which are arranged in a partial tandem and inserted into a single-copy non-LTR retrotransposon. Such partial tandems can be transpositionally active, as was demonstrated by the successful introduction of a similarly structured *Penelope* copy from *D. virilis* into *D. melanogaster*[17]. Since the structure of another non-LTR retrotransposon closely related to the *Pen2* target (76% identity) was already known, it was possible to determine that the *Pen2* insertion in this case did not cause a TSD, but instead formed its 5′ junction *via* microhomology-mediated annealing, as was described for long interspersed elements (LINEs) [18]. Its 193-bp pLTR contains an 18-bp palindrome at the 5′ end. This copy may also have given rise to another insertion on scaffold 304 organized in a way identical to *Pen2a*, that is, flanked by inverted pLTRs with a 10-bp TSD (Figure 2c). This incomplete derivative contains an internal microhomology-mediated deletion and an in-frame stop codon. A related *Pen2* subfamily consists of two 5′-truncated members, with the longest one containing an in-frame stop codon and flanked by inverted 212-bp pLTRs, but no TSD.

*Pen1_Av* elements exhibit a similar overall structure to *Pen2_Av*, except that, in most cases, pLTRs are present in direct orientation only. Unexpectedly, the 3′-terminal pLTR in *Pen1* is separated from the rest of the element by a long unique spacer varying between 0.9 and 1.5 kb in length (Figure 2e, f). Apparently, the spacer is formed via capture of host DNA, as copies that are 90% identical in sequence exhibit no detectable nucleotide sequence similarity between spacers. This family contains the largest number of apparently intact copies: three out of four members code for an 830-aa ORF preserving all of the functionally conserved characteristic RT and EN motifs.

**Figure 3:**
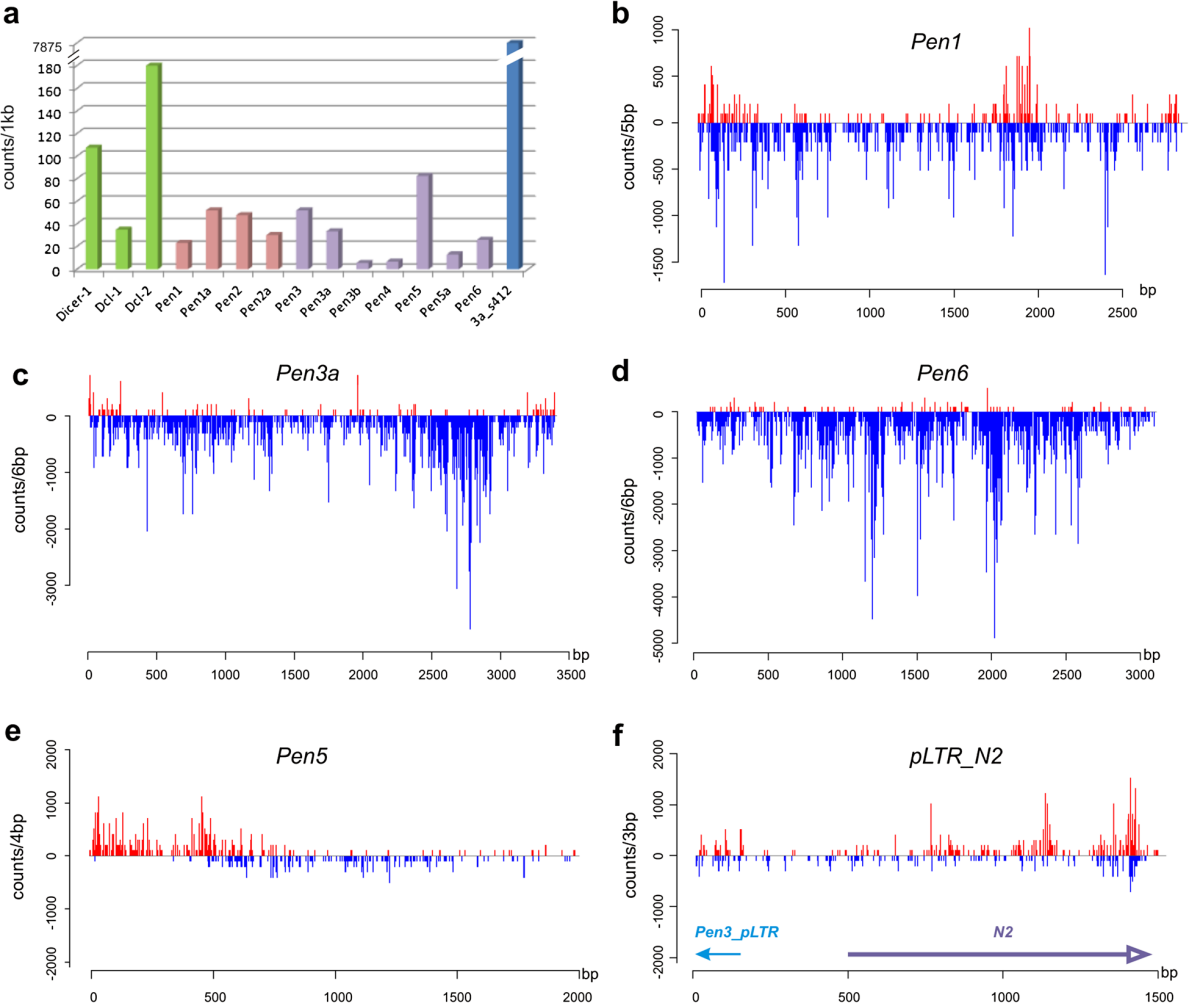
**Transcription and RNA-mediated silencing of*A. vaga Penelope*families. (a)** RNA-seq counts per family. Numbers on the Y axis represent RNA-seq counts per kb, and the moderately transcribed *A. vaga* Dicer-like genes [14] are used for comparison. Transcripts originating from the N2-related segment on scaffold 412 (see text) could not be accommodated using the same scale. **(b-e)** Small RNA coverage plots along selected full-length *Penelope* copies shown in Figure 2. X axis, element length in bp; Y axis, small RNA counts per window of indicated size; red, sense reads; blue, antisense reads. **(f)** Coverage plot for scaffold 412; *Pen3A* pLTR is in the opposite orientation to the transcribed N2-related segment.

*Pen5_Av* and *Pen6_Av* elements are transpositionally inactive, as even their longest representatives contain internal deletions, in-frame stop codons or 5′-truncations. In two cases, ancestral *Pen5* and *Pen6* insertions apparently became fixed as two allelic versions: these 5′-truncated copies are surrounded by the same host genes with an overall 5% sequence divergence across the entire locus (Figure 1). A similar pattern is seen for two allelic versions of a highly decayed *Pen1* copy on scaffolds 19 and 160. *Pen5_Av* and *Pen6_Av* are bounded by inverted pLTRs; however, their exact boundaries are more difficult to determine because of possible decay. Interestingly, despite the presence of two stop codons and a frameshift, a divergent *Pen5* copy is actively transcribed, as evidenced by RNA-seq data (see below, Figure 3a), and contains a spliceosomal intron within the ORF.

### Asparagine-rich insertions in *Penelope* coding sequences

An early-branching lineage of *Pen2a,* represented by two copies in the assembly, contains a lengthy 1.5-kb insertion (N1) into its coding region between the RT and EN domains (Figures 1 and 2d). The inserted fragment is exceptionally rich in asparagine (N) residues (approximately 30%), but nevertheless it does not interrupt the ORF on scaffold 942, resulting in a 1,309-aa coding sequence. Part of the N-rich insert (304 bp) is also found upstream of the 5′ pLTR in inverted orientation (Figure 2d).

Analogous structural variations were observed in *Pen3* and *Pen4* families, albeit in a different relative position - between the core RT and the thumb domain (Figure 2g-i). Due to the tendency to form long tandem/inverted repeat structures, none of these elements could be initially assembled in its entirety, with the exception of one copy on scaffold 607 containing an in-frame stop codon (Figure 2g). Two *Pen3* copies were located on detached contigs containing permuted overlapping 5′- and 3′-terminal parts of the element, in inverted orientation. In these cases, the original structure was inferred by reconstituting the entire ORF from its overlapping N- and C-terminal halves, so that it would be similar in structure to the copy shown in Figure 2g. However, such structures cannot be dismissed as assembly artifacts, because small RNA profiles (see below) switch polarity between the two halves. All *Pen3* and *Pen4* elements carry an insertion of different N-rich segments (N2 to N4) into an ancestral copy resembling a single-copy intact *Pen3a*, which has no such insertions (Figure 1, Figure 2i). There were at least three independent occurrences of long N-rich insertions into the same relative position of *Pen3/Pen4*, with the length of inserted segments varying between 460 and 554 aa, all of them having an asparagine content of 25 to 30%. Overall, it appears that N-rich insertions tend to arise in ORF regions that were initially enriched in short (AAY)_n_ motifs.

### Transcription and RNA-mediated silencing

We sought to determine the possible origin of the N-rich segments inserted into *Penelope* ORF. While the 1.5-kb segments in *Pen2a* could not be found elsewhere in the assembly without being connected to *Penelope* ORF, a 1-kb region of homology 85% identical to the N-rich segment from *Pen3* was found near a *Pen3* solo pLTR on scaffold 412, and, moreover, it was highly transcribed on its own, but yielded a very poor antisense small RNA signal (Figure 3a, f). In contrast, transcription from most individual *Penelope* families was more than an order of magnitude lower, and was comparable in intensity to weakly transcribed *A. vaga* genes, such as *Dicer* endonuclease homologs [14] (Figure 3a). Families without full-length copies, such as *Pen3b* and *Pen4*, yielded very low transcript levels close to background, while *Pen5* yielded higher transcript levels and low antisense small RNA coverage (Figure 3e). Overall, most *Penelope* families exhibit relatively low RNA-seq coverage and high steady-state levels of endogenous small RNA coverage predominantly in antisense orientation, indicating efficient operation of RNA-mediated silencing mechanisms directed against their activity (Figure 3).

### Role of *Penelope* in host gene expansion

**Figure 4:**
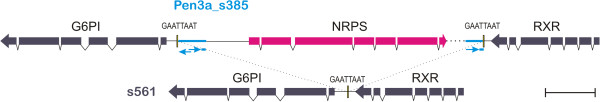
***Penelope*-mediated mobilization of an intron-containing NRPS gene.** Shown are the two scaffolds which constitute an allelic pair containing glucose-6-phosphate isomerase (*G6PI*) and RXR-like retinoic acid receptor (*RXR*) coding sequences. Scaffold 385 harbors a NRPS insertion flanked by *Pen3a* pLTRs (see Figure 2i) and by an 8-bp target site duplication (GAATTAAT), which is present only once on scaffold 561. Introns are denoted by V-shaped lines; other features are as in Figure 2. Scale bar, 1 kb.

The single-copy *Pen3a_Av* (Figure 2i), which may have lost its mobility due to a *pogo* transposon insertion at the very end of the 3′ pLTR, may have participated in retrotransposon-mediated host gene family expansion. Its ORF apparently acted *in trans* to yield an integration event in which a gene coding for a non-ribosomal peptide synthetase (NRPS), initially of bacterial origin, was copied into a new chromosomal location (Figure 4). This NRPS gene has preserved all of its introns, which are present in other members of this multigene family (totaling about 50 copies per genome). It is flanked by two direct and one inverted pLTR in an arrangement similar to that depicted in Figure 2a, and by an 8-bp TSD. The pLTR of *Pen3a* has a nearly-perfect 38-bp palindrome at its 5′ end. The NRPS ORF is located between the two direct pLTRs, but there are no internal sequences in common with *Pen3a*, indicating that the *cis*-acting pLTR sequences were sufficient to provide integration of the entire structure in *trans*. We also found a few other cases in which unrelated sequences were captured between solo pLTRs and surrounded by TSDs, although those sequences did not code for proteins. In addition, a more ancient *Pen3*-mediated event may have resulted in transposition of another multigene family member encoding a leucine-rich repeat (LRR) protein: it is present on two allelic scaffolds (875 and 956), with each allele containing the same pLTR to the 3′ end of the LRR gene. Several solo pLTRs were not associated with any other *Penelope* sequences or TSDs, indicating that recombination and/or deletion of surrounding sequences may have played a role in their formation.

To verify that the pLTR-NRPS combination indeed originated as a result of transposition, rather than a recombinational event bringing together two pLTRs with the same adjacent 8-bp sequence by chance, we searched the assembly for the putative “empty site”. Indeed, we found that scaffold 385 has an allelic partner, scaffold 561, containing the same genes (glucose-6-phosphate isomerase and retinoic acid receptor RXR-alpha) with an overall nucleotide sequence divergence of 2% (Figure 4). As expected, the 8-bp TSD is present on scaffold 561 only once, providing direct evidence that it was indeed duplicated upon insertion of the entire pLTR-NRPS structure.

### Phylogenetic placement of *A. vaga Penelope* families

**Figure 5:**
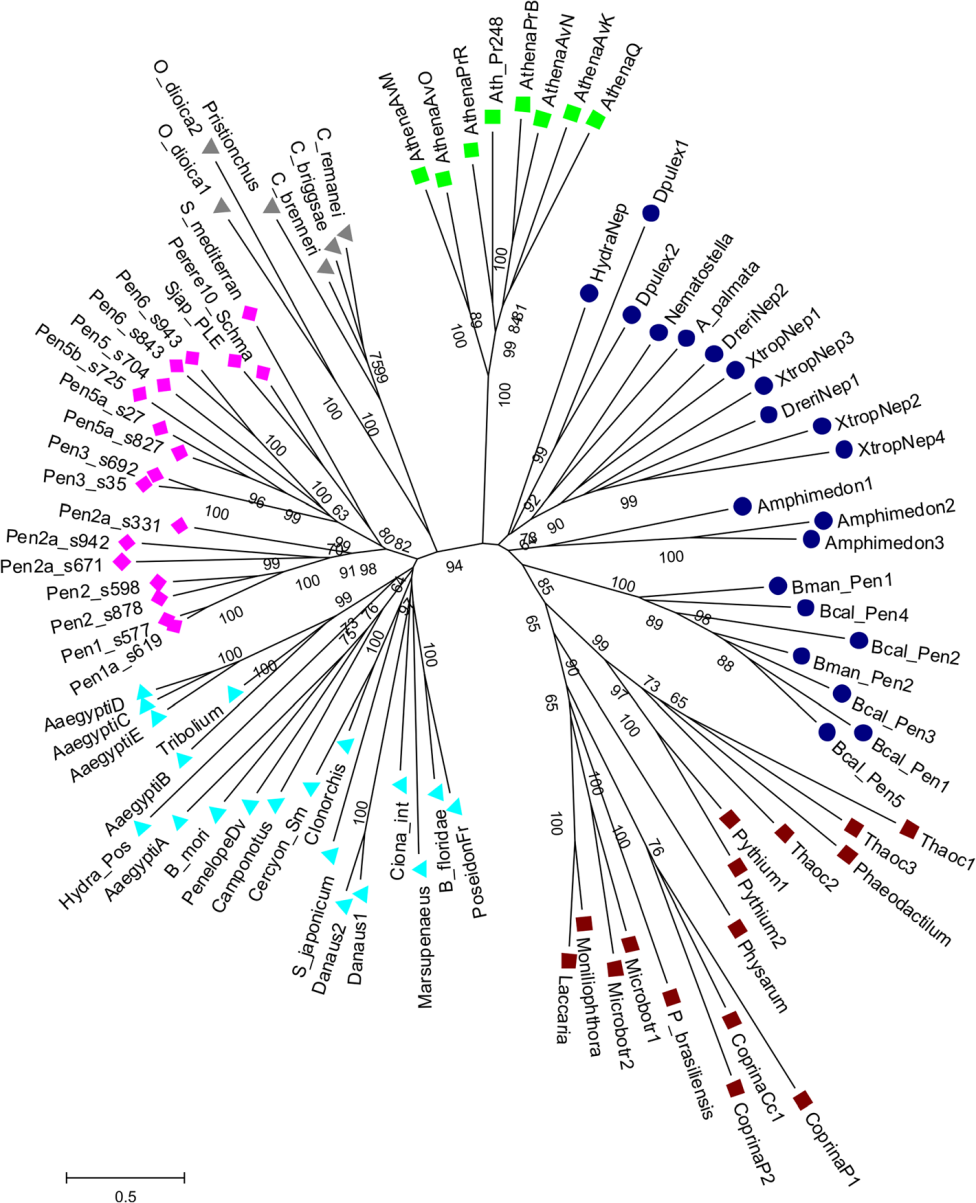
**Phylogenetic relationships of EN-containing and EN-deficient PLEs.** Clade markers are as follows: *Perere* in flatworms and *A. vaga*, magenta diamonds*; Penelope/Poseidon*, cyan triangles; *Neptune*, blue circles; *Nematis*, gray triangles; EN-deficient *Athena*, green squares; EN-deficient protist and fungal PLEs, brown squares. Most taxon designations are from datasets in [10,20]; Bman, *Brachionus manjavacas*; Bcal, *Brachionus calyciflorus*; EN, endonuclease; PLEs, *Penelope*-like elements. Branch support values over 60% are shown. Scale bar, amino acid substitutions per site.

Comparison of *A. vaga Penelope* retrotransposons with PLEs from other taxa shows that they are most closely related to the flatworm *Perere* clade containing *Perere10* from *Schistosoma mansoni, S. japonicum* and *Schmidtea mediterranea*[19,20]. The two *A. vaga Penelope* branches are about as distant from each other as they are from the flatworm *Perere10* elements (Figure 5). They are clearly grouped with other retrotransposons of the *Penelope/Poseidon* group, as opposed to *Neptune*-like retrotransposons, which have a cysteine-rich domain between RT and EN [20]. In this respect, it is worth mentioning that *Pen3_Av* and *Pen5a_Av* families contain a short Cys-rich insert in a different location, between the RT core motifs 3 and 4 (Cys/His-X_1-3_-Cys-X_10_-Cys-*X*_2_-Cys). Its significance, however, remains obscure. Most PLEs that can be found in early genomic drafts of two species of monogonont rotifers, *Brachionus manjavacas* and *B. calyciflorus*, are closely related to the *Neptune* group (Figure 5) and contain the characteristic cysteine-rich domain between RT and EN [20]. In agreement with our earlier findings [10], the most abundant type of *A. vaga* PLEs, the telomere-associated EN-deficient *Athena* retroelements, constitute a distinct clade, as do the EN-deficient PLEs from fungi and protists. It should be kept in mind that the current phylogeny includes only the extant *A. vaga* families, as this species keeps very little “fossil record” of TEs due to actively ongoing deletion and high TE turnover rate as a consequence of desiccation-induced DNA repair and, therefore, any ancestral evolutionary intermediates are likely to have been eliminated from the genome.

## Discussion

Completion of the high-quality draft genome sequence of a bdelloid rotifer, *Adineta vaga*, provides us with an opportunity to investigate the entire TE complement in a long-term asexual species, and to obtain a comprehensive picture of genome-wide TE distribution and evolutionary history. This study is focused on PLEs, an enigmatic class of retroelements which include EN-containing retrotransposons from numerous animal genomes, as well as telomere-associated EN-deficient retroelements from rotifers, fungi, protists and plants [10,16,20]. While we observe co-existence, within the genome of the same species, between the conventional *Penelope* retrotransposons with the GIY-YIG EN domain and the EN-deficient PLEs, as was recently reported in the kuruma shrimp [21], there is no indication of cross-mobilization of EN-deficient *Athena* elements by the *Penelope*-encoded EN. For each *A. vaga Penelope* family, its mobility in the genome apparently relies on the presence of element-specific terminal structures required for retrotransposition, termed pLTRs, which do not exhibit any association with *Athena* elements. It should be noted that fungal genomes contain only EN-deficient PLEs and no EN-containing ones, again indicating that the maintenance of the former does not depend on the latter.

The present analysis of *Penelope* retrotransposons in *A. vaga*, while illustrating their overall similarity to *Penelope* elements in other species, including the extreme structural variability, also highlights their peculiar features that may contribute to the evolutionary plasticity of the bdelloid genome characterized by high levels of gene conversion, by relatively low but highly diversified TE content, and by the presence of numerous genes of foreign origin and substantial lineage-specific expansions of various multigene families [14]. Expansions involve gene families, including NRPS and other foreign genes, as well as 7-transmembrane receptors and proteins containing repeated motifs, such as LRR, TPR, PPR, Kelch, NHL, FG-GAP and so on. Paradoxically, many gene families are amplified to a much higher copy number than TE families. These multigene families are likely involved in processes that involve diversification of gene function, such as host defense and immunity, production of secondary metabolites, chemosensory perception, extracellular signaling and cell-cell communication.

We find that *A. vaga Penelope* elements can mobilize host genes surrounded by terminal pLTR structures and, therefore, can contribute to observed lineage-specific expansions of certain gene families, shedding light on some of the mechanisms that multiply host genes to copy numbers higher than most TEs. While other TE classes also have the potential to contribute to amplification of gene families, which could then be followed by their diversification, *Penelope* elements have a distinct advantage over other retrotransposons in this respect, as their retrotransposition mechanism apparently allows intron retention [1]. Our analysis reveals no strong evidence that any intact *A. vaga Penelope* ORFs were exapted as domesticated genes, as none of them are present on two collinear allelic chromosome segments. Those *Penelope* fragments that we do find in collinear pairs are badly damaged, and their function, if any, would not involve *Penelope*-encoded products. The most likely agents involved in gene amplification are the *Penelope* families with the capacity to incorporate relatively long stretches of host DNA between pLTRs, such as *Pen1_Av* and *Pen3a_Av*. Four out of six apparently intact *Penelope* ORFs belong to these families. While the propensity of *A. vaga* for DNA deletion could rapidly erase one or both pLTRs from the genome, making it difficult to detect additional cases of pLTR-mediated gene amplification, the example described here leaves little doubt that such events can indeed contribute to lineage-specific expansion of multigene families.

Even though the overall TE content in *A. vaga* is quite low by metazoan standards, the particularly low *Penelope* copy number in comparison to other retrotransposons is striking. While some TEs could remain undetected in a *de novo* assembly consisting of over 30,000 scaffolds with N50 of 260 kb [14], there is little reason to believe that most *Penelope* copies would be preferentially undetectable. Two circularly permuted copies located on isolated contigs with little or no flanking sequences may represent active elements which could not be properly assembled due to the fusion of several identical copies into a single contig. Although studies of PLE distribution along the chromosome length will have to await chromosome-sized scaffolds, the majority of *Penelope* copies are present on relatively small scaffolds (Additional file 1: Table S1), and inspection of their genomic environment shows that they are largely compartmentalized in TE-rich regions, which may represent non-essential genomic islands consisting of various TEs, genes of foreign origin and members of diverse multigene families.

Like all other TEs in the genome, *Penelopes* are subject to the generalized host defense responses, such as RNA-mediated silencing. Indeed, we find that most of the *A. vaga Penelope* copies give rise to small RNAs with preferential antisense polarity. The *Penelope* element in Drosophila was previously shown to elicit small RNA response after invasion [22,23]. We also observed that many *Penelope* copies were disabled by microhomology-mediated deletions, a mechanism of TE inactivation that is applicable to most other TEs and likely operates during DNA repair following frequent cycles of desiccation and rehydration [14,24]. However, *Penelope* elements constitute only about 2% of *A. vaga* TEs, and only 4% of its retroelements. Thus, additional family-specific mechanisms should be invoked to explain their much lower relative abundance in comparison with other TEs. Most likely, their low proliferation capacity may be associated with peculiarities of their replication mechanism in this species.

A previously undescribed phenomenon is the appearance of very long inserts in the coding regions of *Pen2-Pen5* elements, which do not necessarily disrupt ORF integrity and are highly enriched in asparagine residues. It appears that copies with such inserts would still be capable of retrotransposition, although their ORFs would be increased in size from the usual 800 to 900 to 1,300 to 1,500 aa, and the domain structure perturbed. For *Pen2a*, the inserted segment could serve as a long linker between the RT and EN domains, while in *Pen3 to 4* such a linker would connect the core RT with its thumb domain. Analogous inserts have not been previously observed in other TEs, and it is reasonable to suggest that they arise as a consequence of the complicated molecular gymnastics that PLEs perform during their replication. In particular, the existence of an autonomous highly transcribed N-rich segment in the vicinity of *Pen3a* pLTR indicates that it could have been captured *in trans* and internalized. We also noticed that in the candidate precursor elements, such as *Pen2* and *Pen3a,* regions roughly corresponding to the linker insertion sites in *Pen2a* and *Pen3 to 4* contain several short stretches of asparagine residues. In addition, a secondary insertion of *Pen2a* into *Pen3* on scaffold 671 also occurred into the N-rich segment, indicating that this sequence may serve as an attractive target for *Penelope* insertions. Since *Penelope* elements were previously reported to favor simple AT-rich sequences as preferred targets [16], we propose that such inserts may arise as a result of spurious self-priming by read-through transcripts containing the adjacent flanks enriched in simple trinucleotide repeats, followed by template jumps. In such cases, the short internal stretches of the N-rich coding sequence (AAT)_n_ or (AAC)_n_ could help in keeping the reading frame of the inserted segment properly aligned, and a chimeric ORF could persist in the genome if it codes for an uninterrupted polypeptide.

The propensity of *Penelope* elements for self-priming may be inferred from the abundance of inverted-repeat structures containing palindromes at the inverted junction, as shown in Figure 2. Consistent occurrence of such palindromes is best interpreted in terms of self-priming, which, however, would have to occur on an antisense template (if a sense template is used, self-priming at the 3′ end would result in a tail-to-tail inverted repeat arrangement, as opposed to the most frequently observed head-to-head). Moreover, utilization of an antisense template is highly compatible with intron retention, since introns would not be recognized in an antisense orientation by the splicing machinery. The presence of oppositely oriented promoter motifs in pLTRs also argues in favor of bidirectional PLE transcription. However, we cannot currently exclude the possibility of utilization of an unspliced sense transcript as a template, and further experiments will be required to discriminate between these possibilities. Direct demonstration of antisense promoter activity in *Penelope* elements and full elucidation of its replication cycle constitutes a promising subject for future studies.

## Conclusions

*Penelope* elements occupy a special place among TE superfamilies because of their variable structure representing a flexible arrangement of direct and/or inverted repeats. This structure exhibits a high degree of conservation among all animals harboring EN-containing PLEs, while the protist and fungal genomes contain only EN-deficient PLEs, which do not share this structural organization. Our analysis reveals co-existence of two distinct PLE types within the genome of the same host species with no evidence of cross-mobilization between families, indicating that the element-encoded enzymatic activities and its *cis*-acting sequences are co-adapted. The EN-containing PLEs were shown to participate in expansion of intron-containing multigene families in the host. We also describe a new phenomenon of insertion of long N-rich segments into the coding sequence, not previously observed in other retroelements, and hypothesize that it may occur as a consequence of the atypical replication mechanism. Taking all of the observed structural features into consideration, we hypothesize that EN-containing PLEs use a self-priming mechanism of replication, which would result in intron retention if it utilizes an antisense template. However, further experiments are required to discriminate between possible alternative models.

## Methods

Initial PLE identification in *A. vaga* was done in the course of genome analysis as described in [14], with relatively low recovery from REPET and ReAS pipelines due to the low copy number, which hampers identification of numerous single-copy elements. Additional BLAST searches were performed using the conserved GIY-YIG and RT domains as queries, and after identification and boundary adjustment of full-length copies, these were used as queries to identify shorter fragments using BLAT [25]. Multiple sequence alignment was done with MUSCLE [26]. *A. vaga Penelope* sequences were aligned as amino acids in MEGA v.5.10 [27] and untranslated back into nucleotides. Maximum likelihood analysis of nucleotide sequences was performed with RAxML [28]. For comparison with PLEs from other species, the initial datasets from [20] and [10] were supplemented with PLEs characterized in the present study, and neighbor-joining and minimum evolution analyses of protein-coding sequences were performed in MEGA (Poisson model, gamma distributed rates among sites, 1,000 bootstrap replications). RNA-seq counts were determined with the aid of a custom Ruby script available upon request. DNA and RNA sequencing data were generated by the *A. vaga* sequencing consortium, and all of the scaffold numbers and coordinates correspond to the assembly in [14]. Validation of selected sequences and closure of gaps in genomic DNA was done by PCR using custom oligonucleotide primers available upon request. Detailed procedures for small RNA isolation and analysis will be published elsewhere (Rodriguez and Arkhipova, in preparation). Briefly, HiTrap Q (GE Healthcare Life Sciences, Pittsburgh, PA, USA) column chromatography eluates corresponding to protein-bound fractions from *A. vaga* lysates were collected to extract endogenous small RNAs as described in [29]. This protocol results in preferential extraction of small RNAs bound to Argonaute/Piwi proteins, and over 80% of the obtained reads correspond to the piRNA-like category 25 to 32 nt in length with a strong 5′-uridine bias. A small RNA library was processed for sequencing on the Illumina HiSeq platform. After initial filtering, small RNAs were mapped using Bowtie [30] to the individual *A. vaga Penelope* sequences. Coverage plots along each element were produced with custom C and R scripts.

## Abbreviations

bp: Base pair(s); EN: Endonuclease; Inr: Initiator; LRR: Leucine-rich repeat; NRPS: Non-ribosomal peptide synthetase; ORF: Open reading frame; pLTR: Pseudo-LTR; PLE: *Penelope*-like element; PCR: Polymerase chain reaction; RT: Reverse transcriptase; TE: Transposable element; TSD: Target site duplication.

## Competing interests

The authors declare that they have no competing interests.

## Authors’ contributions

IA designed the study, performed data analysis and wrote the manuscript. IY compiled the TE inventory, analyzed transcription data, validated genomic sequences by PCR and closed the sequence gaps. FR constructed small RNA libraries and participated in data analysis. All authors read and approved the final manuscript.

## Supplementary Material

Additional file 1: Table S1 Inventory of the *A. vaga Penelope* full-length copies and fragments in the assembled scaffolds. Click here for file

Additional file 2 **Nucleotide sequence alignment of *A. vaga Penelope* coding sequences shown in Figure **1**.** Click here for file
